# Short-term high sodium intake increases nocturnal blood pressure but not arterial stiffness in Black adults

**DOI:** 10.1007/s00394-026-03994-w

**Published:** 2026-05-26

**Authors:** Stacy D. Hunter-Cooper, Mitra Rahimi, Pierrick Millet, Micqauella R. Lopez, Stavros A. Kavouras, Philimon N. Gona

**Affiliations:** 1 Cardiovascular Physiology Laboratory, Department of Health & Human Performance, Texas State University, San Marcos, TX 78666 USA; 2 Hydration Science Lab, College of Health Solutions, Arizona State University, Phoenix, AZ USA; 3 Department of Urban Public Health, Manning College of Nursing and Health Sciences, University of Massachusetts Boston, Boston, USA

**Keywords:** Sodium intake, Blood pressure, Arterial stiffness, Hypertension

## Abstract

**Introduction:**

High sodium intake has been associated with greater pressor responses in Black compared with White adults. While the acute effects of sodium on BP have been studied in Black adults, to our knowledge, no studies have explored sodium’s effects on arterial stiffness in this population.

**Purpose:**

This study investigated the effects of short-term high sodium intake on BP and arterial stiffness in Black men and women. Urinary and blood markers associated with renal sodium handling were also assessed. We hypothesized that high-sodium intake would be associated with increased BP and arterial stiffness.

**Methods:**

Thirty-four participants (ages 20–60 years) completed a dietary intervention consisting of 3 days of low-sodium intake (≤ 31 mmol/day) followed by 3 days of high-sodium intake (201 mmol/day). Ambulatory BP and 24-h urinary sodium excretion were measured during the final 24 h of each dietary phase and cardio-ankle vascular index (CAVI), urine specific gravity, and hematocrit were measured under fasting conditions the mornings after each 3-day dietary phase.

**Results:**

Statistical analysis revealed that three days of high-sodium intake was associated with significant increases in body mass, nighttime diastolic and mean BP, and urinary sodium and sodium excretion compared to low-sodium intake (*P* < 0.05 for all). Hematocrit was significantly reduced after the high-sodium dietary intervention (*P* < 0.001). Arterial stiffness as measured via CAVI was unaltered.

**Conclusion:**

In summary, short-term high sodium intake resulted in increased nocturnal BP without significant changes in arterial stiffness in Black men and women.

## Introduction

Sodium overconsumption is common in the U.S. and when practiced long term, associated with increased risks of hypertension (HTN), stroke, and all-cause mortality [1, 2]. Brief periods, e.g., a hypertonic saline infusion or 5–7 days of high sodium intake also pose negative effects increasing blood pressure (BP) in salt-sensitive adults while eliciting acute vascular dysfunction in salt-sensitive and salt-resistant individuals [3–7]. African Americans are disproportionately affected by some of these effects exhibiting greater BP elevations following high sodium infusion [6] or dietary intake [8] along with greater prevalence of salt sensitivity in general [8] when compared to their White counterparts. Mechanisms accounting for racial discrepancies in the hemodynamic responses to high sodium intake are not fully understood though speculated upon in a recent review [9].

Studies demonstrating vascular dysfunction following high sodium intake have primarily focused on endothelial function [10]. Recent findings from our group showed that 3 days of high sodium intake tended to reduce flow-mediated dilation (FMD) in Black women [11]. This finding is consistent with previous results showing declines in FMD following 5–7 days of high sodium intake in predominantly White and Asian adults [7, 12]. Relatively few studies have investigated sodium’s effects on arterial stiffness. A positive correlation between sodium intake and arterial stiffness has been demonstrated [13] and 7 days of sodium loading increased heart-femoral pulse wave velocity (PWV) in salt-sensitive but not salt-resistant adults [14]. While high sodium intake increased carotid-femoral PWV in middle-aged adults, this increase in PWV was no longer significant after adjustment for mean arterial BP [15]. These results suggest that short-term sodium-induced alterations in arterial stiffness could be driven by increases in BP.

Cardio-ankle vascular index (CAVI) is an index of central and peripheral arterial stiffness associated with increased CVD events [16]. This index of arterial stiffness applies Bramwell-Hill’s stiffness parameter β equation which, based on mathematical computation, is theoretically not influenced by BP at the time of the measurement [17]. Studies have supported this theory showing that CAVI, unlike PWV, is unaltered in the presence of changes in BP [18–20]. Thus, investigating the effects of high sodium intake on CAVI could yield insights into potential BP-independent effects of high sodium intake on the vasculature.

To our knowledge, the effects of high sodium intake on CAVI have not been previously assessed. Moreover, the effects of high-sodium intake on arterial stiffness have not been established in African American adults. Therefore, the primary aim of this study was to investigate the effects of short-term high sodium intake on CAVI in African American/Black men and women. Our hypothesis was that arterial stiffness would increase after sodium loading in this population. We further examined the effects of sodium loading on ambulatory BP and blood and urinary indicators of renal sodium handling. Findings from this investigation could shed light on BP-independent adverse effects of high-sodium intake and facilitate a greater understanding of the mechanistic underpinnings of the relationship between salt intake and CVD risk.

## Materials and methods

### Study population

Study procedures were approved by the Texas State University Institutional Review Board (Approval Number: 8663) and were performed according to the 1964 Declaration of Helsinki ethics standards. Informed written consent was also obtained from all participants before data collection. African American/Black, men and women between the ages of 20 and 60 years were recruited via flyers and university-wide emails. Exclusion from participation was based on the following criteria: i) pregnancy or within 60 days postpartum; ii) recent use of blood pressure, statin, or ACE inhibitor medications within the past 3 months; iii) history of infection (viral or other) within the past 4 weeks; iv) adrenal or endocrine tumors; v) renal disease, defined as a glomerular filtration rate (GFR) less than 60 ml/min/1.73m^2^; vi) prior myocardial infarction; vii) known coronary heart disease; viii) personal history of stroke; ix) heart failure; x) uncontrolled cardiac arrhythmias; xi) recent chest pain or dyspnea; xii) current insulin-dependence; xiii) undergoing chemotherapy or radiation; & xiv) systolic or diastolic blood pressure exceeding 149 mm Hg or 99 mm Hg, respectively. The study protocol was indexed on clinicaltrials.gov (NCT05815043).

### Study design

The current study deployed a nonrandomized, crossover trial design. Descriptive, BP, and urinary and blood marker data during low- and high-sodium conditions from 14 participants have been previously published [11]. Participants completed a familiarization and screening visit consisting of three seated BP measurements (to ensure participants met BP inclusion criteria) along with height, weight, and estimated glomerular filtration rate (eGFR) assessments. Blood samples were obtained via venipuncture and shipped to LabCorp for eGFR analysis using currently recommended procedures. Additionally, participants completed a health history questionnaire and were given low-sodium diet instructions composed by a registered dietitian. Instructions consisted of sample low-sodium recipes, guidance on reading food labels, and lists of high-sodium foods to avoid and low-sodium food options to incorporate into their diets.

Low- and high-sodium diet conditions were completed in succession and participants were instructed to adhere to a 31-mmol-per-day (720 mg per day), low-sodium diet for a total of 6 days. During the high-sodium condition, on days 4–6, participants supplemented their diets with 10 salt tablets per day resulting in a total sodium intake of 201 mmol/day (4660 mg/day). This sodium quantity falls within the range of typical American consumption [21] and has previously been demonstrated to increase BP and reduce endothelium-dependent vasodilation [7]. Participants were instructed to avoid exercise during the 6-day diet and to refrain from ingesting caffeinated beverages ≥ 12 h before the CAVI measurements on days 4 and 7. It has been demonstrated that menstrual cycle phase has no effect on sodium-induced changes in BP [22]. Thus, menstrual cycle phase was not standardized between participants.

### Measurements on days 3 and 6

#### Ambulatory blood pressure

Ambulatory BP monitoring (ABPM) was completed during the final 24 h of each dietary condition (days 3 and 6) using the TM-2441 BP monitor (A&D Engineering, San Jose, CA). Arm measurements were obtained to ensure proper cuff size selection and blood pressure measurements were obtained at 30 min intervals from 7am to 11 pm and hourly between 11 pm and 7 am. Participants were instructed to maintain their normal, daily activities, but to avoid exercising while wearing the device. In accordance with the Canadian Hypertension guidelines, a minimum of 20 daytime and 7 nighttime measurements was required for validity [23].

#### 24-hour urinary sodium excretion

During the final 24 h of the low- and high-sodium dietary conditions, urine samples were collected using one-gallon containers. Urine collection containers were weighed for urine volume and urine aliquots were assessed for urine specific gravity and sent to LabCorp for the measurement of urinary sodium via ion selective electrode. Urinary sodium concentrations and 24-h urine sample volumes were used to determine 24-h sodium excretion during each dietary phase.

### Measurements on days 4 and 7

#### Blood pressure

Seated BP was measured in the mornings after each dietary condition prior to blood draws using the TM-2441 BP monitor (A&D Engineering, San Jose, CA). The same cuff previously sized for ambulatory BP measurements was used to obtain this measurement in the lab. Blood pressure was measured after 5 min of seated rest and the averages of 3 measurements were recorded.

#### Hematocrit

On days 4 and 7 (after each dietary condition), participants visited the laboratory in the morning after having fasted overnight. An antecubital blood draw was performed to obtain a whole blood sample for hematocrit determination via microhematocrit centrifugation.

#### Cardio-ankle vascular index

Cardio-ankle vascular index measurements were completed at the same time of day ± 1 h (to minimize the influence of diurnal variations) using the Vasera VS-1500 (Fukuda Denshi, Redmond, WA). During this procedure, simultaneous brachial and ankle pressure waveforms, electrocardiogram (ECG), and phonocardiogram were recorded while subjects lay in the supine position. CAVI was measured unilaterally on the left side and determined via automated computation using the below formula where a and b are constant values determined by the device to match aortic PWV, ρ is blood viscosity, and ΔP = Systolic BP – Diastolic BP [24].

$$ {\mathrm{CAVI}} = {\mathrm{a}}\left[ {\left( {{2}\rho /\Delta {\mathrm{P}}} \right) \times {\mathrm{ln}}\left( {{\mathrm{Systolic}}\;{\mathrm{BP}}/{\mathrm{Diastolic}}\;{\mathrm{BP}}} \right){\mathrm{PWV}}^{{2}} } \right] + {\mathrm{b}} $$

**Fig. 1:**
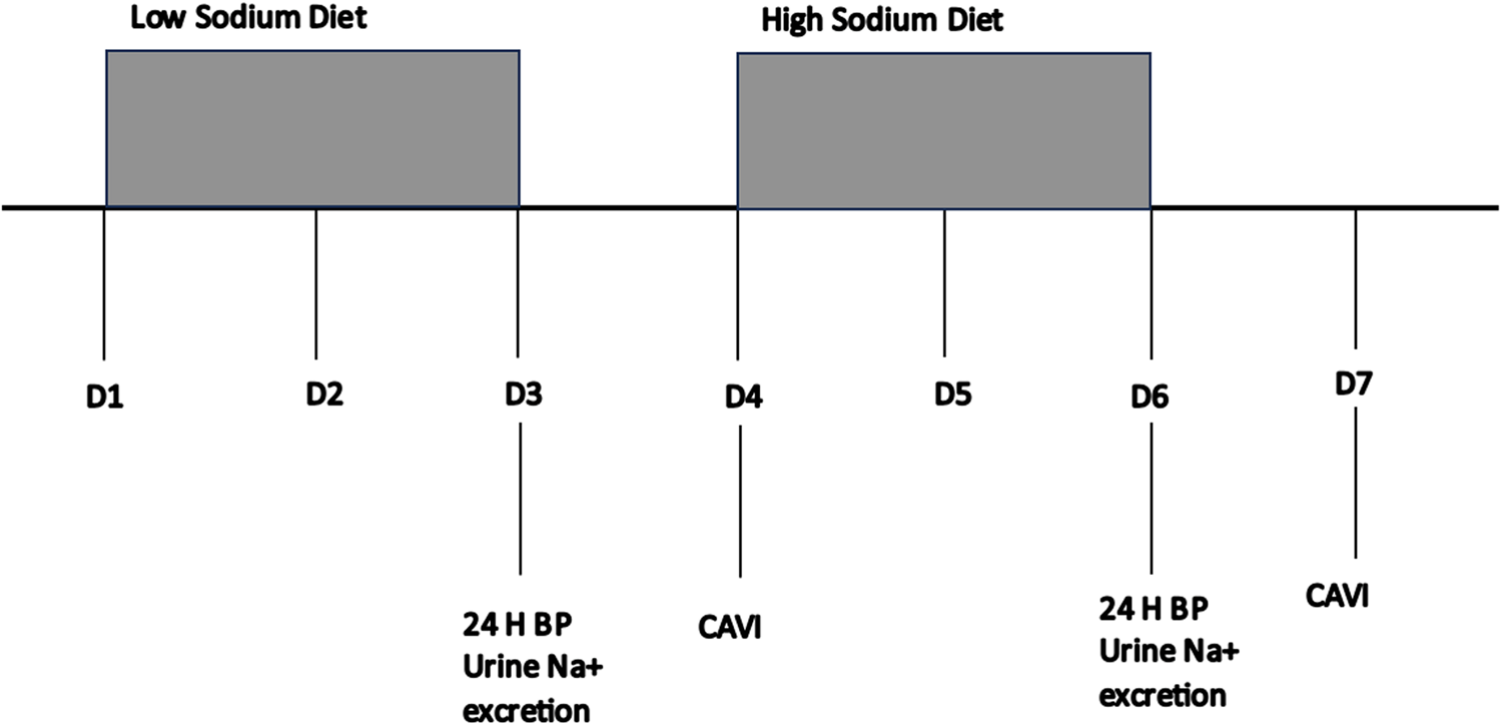
Procedure Flowchart. After eGFR and blood pressure screening, participants adhered to low- and high-sodium diets for 3 days each. Ambulatory blood pressure monitoring and urinary sodium excretion were assessed. Cardio-ankle vascular index (CAVI) was measured in the mornings after each 3-day dietary phase

The average of two measurements is reported. Study procedures are summarized in Fig. 1.

### Statistical analyses

Normality of quantitative variables was assessed using the Shapiro–Wilk test. With the exception of hematocrit, all quantitative dependent variables were normally distributed. These variables were summarized using the means and standard deviations. Paired samples t-tests were used to compare low- vs. high-sodium conditions. For hematocrit, due to the veracity of the normality assumption having been violated, the nonparametric Wilcoxon Signed Rank test was used to compare low- and high-sodium conditions. Statistical significance was set a priori at *P* ≤ 0.05.

## Results

### Demographic data

Of the 75 volunteers prescreened, 44 met the inclusion criteria, of which 10 declined to participate or were lost to follow up, resulting in a final sample of 34. All but four participants had Black paternal and maternal parents. Four participants identified as being multiracial: 2 females identified as Black and Hispanic/Latinx and one female and one male participant identified as both Black and White.

Baseline subject characteristics are outlined in Table 1. Participants were predominantly female (70%), and all were between the ages of 20 and 56 years (mean age 30 ± 11 years). One female participant was postmenopausal but not undergoing hormone therapy. Three female participants were taking oral contraceptive medications at the time of enrollment. Two participants (1 female; 1 male) were switched from salt tablets to ingesting two teaspoons of table salt on day 3 due to reports of nausea. Both participants reported a dissolution of symptoms upon switching and both completed all study follow-up visits.

**Table 1:** Subject characteristics

Sex (Female/Male)	24/10
Age (years)	30 (11)
Height (cm)	169.7 (11)
Body mass (kg)	85.5 (20)
BMI (kg/m^2^)	30.1 (9.2)
eGFR	106 (20)
Seated systolic BP (mmHg)	127 (11)
Seated diastolic BP (mmHg)	82 (10)

Data are presented as means (SD), BMI = body mass index, eGFR = estimated glomerular filtration rate; BP = blood pressure

### Body mass, urinary sodium excretion, and urine specific gravity

Results from low- and high-sodium dietary conditions are displayed in Table 2. A significant main effect of diet was observed for body mass (means ± standard deviations = 85.5 ± 20 vs 86.5 ± 20 kg; 95% confidence interval = −1.4, −0.5; *P* < 0.001), hematocrit (40 ± 4 vs 38 ± 4%; 1.5, 4.3; *P* < 0.001), urine specific gravity (1.012 ± 0.005 vs 1.014 ± 0.007; −0.004, −0.0005; *P* < 0.05), urinary sodium (74 ± 56 vs 150 ± 76 mmol/L; −104, −50; *P* < 0.001), and 24 h sodium excretion (60 ± 47 vs 163 ± 124 mmol/24 h; −145, −58; *P* < 0.001). Compared to low-sodium conditions, 3 days of sodium loading was associated with statistically significant increases in body mass, urine specific gravity, and urinary sodium and 24 h sodium excretion. In addition, hematocrit was significantly reduced after the sodium loading condition (40 ± 4 vs 38 ± 4%; 1.5, 4.3; *P* < 0.01).

**Table 2:** Cardiovascular responses and urinary sodium excretion in response to low- and high-sodium conditions in black adults

	Low-Sodium	High-Sodium	*P*-value	Effect size
Body mass (kg)	85.5 (20)	86.5 (20)†	0.0001	−0.76
Office systolic BP (mm Hg)	120 (12)	126 (14)†	0.0003	−0.69
Office diastolic BP (mm Hg)	78 (7)	81 (10)*	0.035	−0.38
Office mean arterial BP (mm Hg)	92 (8)	96 (10)**	0.003	−0.55
24 h systolic BP (mm Hg)	120 (12)	120 (11)	0.795	−0.06
24 h diastolic BP (mm Hg)	75 (9)	75 (9)	0.402	−0.20
24 h mean arterial BP (mm Hg)	89 (10)	90 (10)	0.572	−0.13
Daytime systolic BP (mm Hg)	124 (12)	122 (10)	0.507	0.16
Daytime diastolic BP (mm Hg)	79 (9)	77 (8)	0.197	0.32
Daytime mean arterial BP (mm Hg)	95 (9)	92 (8)	0.151	0.35
Nighttime systolic BP (mm Hg)	111 (11)	113 (14)	0.228	−0.295
Nighttime diastolic BP (mm Hg)	66 (7)	69 (11)*	0.045	−0.51
Nighttime mean arterial BP (mm Hg)	82 (9)	86 (12)*	0.041	−0.54
Hematocrit (%)	40 (4)	38 (4)**	0.0006	0.81
24 h urine specific gravity	1.012 (0.005)	1.014 (0.007)*	0.015	−0.46
Urine Na^+^ (mmol/L)	74 (56)	150 (76)†	0.00002	−1.03
24 h urinary Na^+^ excretion (mmol/24 h)	60 (47)	163 (124)†	0.00004	−0.84

Values are presented as means (SD). *P ≤ 0.05 vs. low sodium. **P ≤ 0.01 versus low sodium. †*P* ≤ 0.001. BMI = body mass index; BP = blood pressure; h = hour

### Office and ambulatory blood pressure and arterial stiffness

**Fig. 2:**
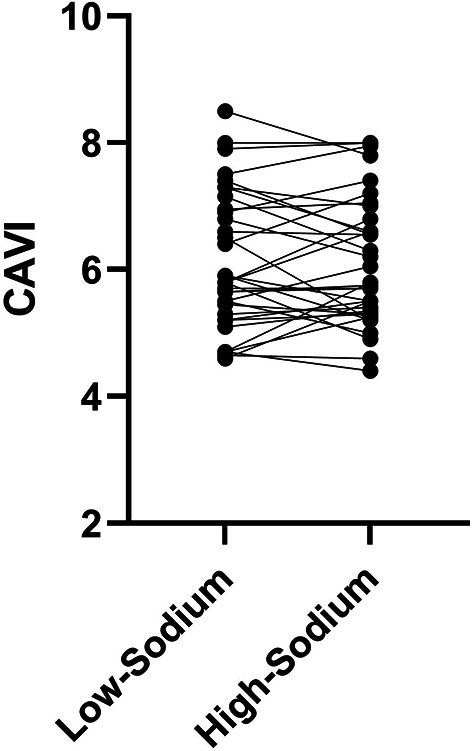
Cardio-ankle vascular index (CAVI) results measured after 3 days of low- and high-sodium intakes are shown in this figure (n = 34). No influence of diet was observed for CAVI

Blood pressure results from measurements obtained in the lab are reported in Table 2. A main effect of diet was observed for systolic (120 ± 12 vs 126 ± 14 mm Hg; −10, −3; *P* < 0.001), diastolic (78 ± 7 vs 81 ± 10 mm Hg; −5, −0.2; *P* < 0.05), and mean arterial BPs (92 ± 8 vs 96 ± 10 mm Hg; −7, −2; *P* < 0.01) with all values significantly elevated after high- versus low-sodium conditions. After excluding ABPM data from participants for whom an insufficient number of BP measurements were obtained, data from 20 participants were analyzed. Though 24 h and daytime BPs were unaltered, sodium loading significantly increased nighttime diastolic (66 ± 7 vs 69 ± 11 mm Hg; −8, −0.1; *P* < 0.05; Table 2) and mean arterial BP (82 ± 9 vs 86 ± 12 mm Hg; − 0.8, − 0.2; *P* < 0.05). No main effect of diet was observed for CAVI (6.1 ± 1 vs 6.1 ± 1; −0.19, 0.23; *P* = 0.568; Fig. 2).

## Discussion

The main findings from the current study indicate that 3 days of high-sodium intake increased nocturnal BP while posing no effects on CAVI in Black adults. Observations indicative of sodium-induced physiological changes included increased body mass, decreased hematocrit, and a pronounced increase in urinary sodium excretion during the high-sodium intake phase.

To our knowledge, this is the first investigation into sodium’s acute effects on arterial stiffness using the CAVI technique. The lack of effect of high-sodium intake on CAVI is consistent with previous findings indicating no effect of high-sodium intake on PWV in salt-resistant [14] and young adults [15]. Though the current study encompassed a wide age range, including both young and middle-aged adults (up to 56 years of age), most participants (85%) were young (between the ages of 20 and 39) which is reflected by the mean age of 30 years. It is possible that young adults are more protected from sodium’s acute vascular effects. It was also demonstrated previously that a high-sodium meal exerted no effects on endothelial function in young adults [25].

Previous studies suggest that the acute effects of high-sodium intake on arterial stiffness are BP-dependent. One study showing an increase in PWV following high-sodium intake in middle-aged adults further revealed that these changes did not persist after adjusting for mean arterial BP [15]. Moreover, 7 days of sodium loading increased heart-femoral PWV only in salt-sensitive but not salt-resistant adults [14]. Whether CAVI, which is based on the beta stiffness parameter [17], is independent of BP has been the subject of scientific debate. Though some evidence exists to the contrary, studies have shown that CAVI is less BP-dependent than PWV [18–20]. Results from the current study concur with this notion given the lack of change in CAVI despite an increase in nocturnal and lab-based mean arterial BPs. While carotid-femoral PWV is the reference standard in the measurement of arterial stiffness, PWV is a BP-dependent measure of arterial stiffness. While CAVI is a more recently developed index of arterial stiffness, its prognostic value has been established in the prediction of cardiovascular-related death and stroke [16].

Alterations in arterial stiffness are mediated by structural and functional changes within the vasculature. While structural changes such as increases in wall thickness and collagen deposition and reductions in elastin, which contribute to arterial stiffening with age, take longer to ensue, functional alterations like changes in sympathetic nerve activity [26] can produce immediate increases in arterial stiffness. Though long-term high sodium intake has been associated with increased arterial stiffness [13], acutely high sodium intake suppresses the activity of the renin–angiotensin–aldosterone system (RAAS). This RAAS suppression could counteract a potential effect of high-sodium intake on arterial stiffness via declines in sympathetic nervous system activity. It has been demonstrated that adults with normotensive and hypertensive BPs exhibit reduced muscle sympathetic nerve activity following 6 days of high sodium intake [27]. This could have accounted for currently observed results. 

The CAVI measurement is theoretically independent of changes in BP; however, according to Shirai et al.[19], while a beta-blocker shown to reduce BP via its effects on cardiac contractility had no effect on CAVI, administration of an alpha receptor blocker known to induce smooth muscle relaxation significantly reduced CAVI in healthy adult men. These results suggest that CAVI is not influenced by changes in BP, but by alterations in vasomotor tone. Increases in vasomotor tone are associated with reductions in endothelium-derived nitric oxide production and reflected by declines in FMD. Several studies have demonstrated reductions in nitric oxide mediated vasodilation following brief periods of high-sodium intake [10]. Interestingly, although FMD tended to decline in Black women in response to the same dietary protocol in our previous work [11], these changes in endothelial function did not translate into increases in CAVI.

The lack of change in CAVI could also have been influenced by changes in plasma volume in response to high-sodium intake. Plasma volume expansion is an immediate result of increased plasma sodium concentrations, a response mediated by aldosterone secretion and vasopressin release. While blood volume was not estimated in the current study, the significant reduction in hematocrit along with the increase in body mass both suggest that plasma volume expansion occurred following the high-sodium condition. As plasma volume expansion can be associated with reductions in vasomotor tone, this effect of high-sodium intake could have provided physiological compensation mitigating the effects of the 3-day high-sodium diet on arterial stiffness in this population. This mechanism is believed to prevent increases in BP in salt-resistant adults and could have prevented more pronounced increases in BP in current study participants.

The lack of change in CAVI could have been influenced by the short duration of the sodium loading protocol (3 days) compared to other trials in which periods of 7 days or longer were used to assess sodium’s influence on arterial stiffness [14, 15]. However, we believe 3 days was a sufficient duration to elicit changes in arterial function as a single high-sodium meal reduced FMD in healthy adults previously [28].

Discrepancies between daytime and nocturnal BP outcomes were observed in the current study as increases in BP were evident during the nighttime measurement timeframe only. This discrepancy speculates the need for a more comprehensive look at ambulatory BP outcomes in response to variations in sodium intake. Indeed, nocturnal ambulatory BP is independently associated with atherosclerotic disease and heart failure [29] and a better predictor of adverse health outcomes when compared to daytime ambulatory BP [30].

The current study being conducted in African Americans is of significance as African Americans are underrepresented in research in general [31]. While African Americans exhibit higher prevalence of salt sensitivity of BP, the vascular effects of sodium in this population remain largely unknown. Speculations as to the influence of race on previous results presents a challenge as many physiology studies have not reported racial demographics of study cohorts. Among studies on sodium’s effects on vascular function, those which reported race were mostly conducted in White [4, 7, 25] or Asian [12, 32] adults.

While no alterations in arterial stiffness were observed in the current trial, the increase in nighttime ambulatory BP following 3 days of high-sodium intake reinforces the importance of limiting sodium intake over the long term. Efforts to restrict sodium intake should be continuous as even brief periods of high-sodium intake are linked with BP changes which, if persistent over time, could result in adverse outcomes.

Strengths of the current trial include the measurement of ambulatory BP which is greatly recommended over office/clinic BP in the management of HTN due to its superior predictive value for cardiovascular mortality [30]. Another strength is the focus on Black adults, a group underrepresented in clinical research and disproportionately affected by salt sensitivity of BP. The assessment of urinary sodium excretion as a measure of dietary compliance also enhanced the rigor of the current study. Limitations include the small sample size, brevity of the dietary manipulation, and a lack of controlled feeding throughout the 6-day diet. Despite this, the almost threefold increase in urinary sodium excretion during the high-sodium intake phase suggests compliance with the study protocol. Furthermore, the lack of randomization of dietary phase could pose an order effect. Despite this, results from this trial are consistent with a previous study [15] in which randomization of diet order was performed.

In conclusion, short-term high sodium intake increased nocturnal BP while posing no effects on CAVI in Black men and women.
